# Supplementary material for: Meiotic divisions and round spermatid formation do not require centriole duplication in mice
Source: PLoS Genet. 2025 Apr 28;21(4):e1011698. doi: 10.1371/journal.pgen.1011698 (PMC12064039; doi:10.1371/journal.pgen.1011698)
Supplement: Attachment — Submitted filename: PGENETICS-D-25-00228_Reviews and Responses - FINAL.docx [file pgen.1011698.s005.docx]

Dear Phil,

Thank you for submitting your manuscript to PLOS Genetics. After careful consideration, we feel that it has merit but does not fully meet PLOS Genetics's publication criteria as it currently stands. Therefore, we invite you to submit a revised version of the manuscript that addresses the points raised during the review process.

Please submit your revised manuscript within 30 days Apr 23 2025 11:59PM. If you will need more time than this to complete your revisions, please reply to this message or contact the journal office at plosgenetics@plos.org. When you're ready to submit your revision, log on to https://www.editorialmanager.com/pgenetics/ and select the 'Submissions Needing Revision' folder to locate your manuscript file.

Please include the following items when submitting your revised manuscript:

* A rebuttal letter that responds to each point raised by the editor and reviewer(s). You should upload this letter as a separate file labeled 'Response to Reviewers'. This file does not need to include responses to formatting updates and technical items listed in the 'Journal Requirements' section below.
* A marked-up copy of your manuscript that highlights changes made to the original version. You should upload this as a separate file labeled 'Revised Manuscript with Track Changes'.
* An unmarked version of your revised paper without tracked changes. You should upload this as a separate file labeled 'Manuscript'.

If you would like to make changes to your financial disclosure, competing interests statement, or data availability statement, please make these updates within the submission form at the time of resubmission. Guidelines for resubmitting your figure files are available below the reviewer comments at the end of this letter.

We look forward to receiving your revised manuscript.

Kind regards,

Paula E. Cohen
Section Editor
PLOS Genetics

Aimée Dudley
Editor-in-Chief
PLOS Genetics

Anne Goriely
Editor-in-Chief
PLOS Genetics

**Dear Reviewers and Editors:**

**Firstly, we would like to thank the reviewers and editors for their time and expert critique of our manuscript. We have addressed all comments. We believe that our manuscript has been made better thanks to addressing your feedback and hope that you will deem it suitable for acceptance at PLOS Genetics.**

**Our responses are highlighted in yellow, and the references to the text and figures are highlighted in green.**

**Journal Requirements:**

1) Please upload all main figures as separate Figure files in .tif or .eps format. For more information about how to convert and format your figure files please see our guidelines: 
https://journals.plos.org/plosgenetics/s/figures

We have uploaded separate .tif files of the figures following the guidelines provided.

2) We have noticed that you have uploaded Supporting Information files, but you have not included a list of legends. Please add a full list of legends for your Supporting Information files after the references list.

We have added the legends of the Supporting Information at the end of the manuscript after the main figure legends. See lines 1107 to 1111.

3) Some material included in your submission may be copyrighted. According to PLOSu2019s copyright policy, authors who use figures or other material (e.g., graphics, clipart, maps) from another author or copyright holder must demonstrate or obtain permission to publish this material under the Creative Commons Attribution 4.0 International (CC BY 4.0) License used by PLOS journals. Please closely review the details of PLOSu2019s copyright requirements here: PLOS Licenses and Copyright. If you need to request permissions from a copyright holder, you may use PLOS's Copyright Content Permission form.
Please respond directly to this email and provide any known details concerning your material's license terms and permissions required for reuse, even if you have not yet obtained copyright permissions or are unsure of your material's copyright compatibility. Once you have responded and addressed all other outstanding technical requirements, you may resubmit your manuscript within Editorial Manager. 
Potential Copyright Issues:
i) Figures 3F, 4A, 5D, and 8. Please confirm whether you drew the images / clip-art within the figure panels by hand. If you did not draw the images, please provide (a) a link to the source of the images or icons and their license / terms of use; or (b) written permission from the copyright holder to publish the images or icons under our CC BY 4.0 license. Alternatively, you may replace the images with open source alternatives. See these open source resources you may use to replace images / clip-art:
- https://commons.wikimedia.org
- <https://openclipart.org/>.

Figures 3F, 4A, 5D, and 8 were all made by hand and, therefore, source images and licenses are not required.

4) Please amend your detailed Financial Disclosure statement. This is published with the article. It must therefore be completed in full sentences and contain the exact wording you wish to be published.

1) State what role the funders took in the study. If the funders had no role in your study, please state: "The funders had no role in study design, data collection and analysis, decision to publish, or preparation of the manuscript."

We state that: “The funders had no role in the study design, data collection and analysis, decision to publish, or preparation of the manuscript.” – See lines 541-542.

2) If any authors received a salary from any of your funders, please state which authors and which funders.

We state that: “Partial salary for MWS was covered by F31HD111265 and R01GM11755. Partial salary for PWJ when at JHU was covered by R01GM11755.” – See lines 540-541.

5) We note that the Data Availability Statement mentioned in the manuscript is different from that provided in the online submission form. The Data Availability statement in the online submission form is currently as follows: 'All data underlying the findings presented in this manuscript will be made fully available, without restriction, upon request.' While the one in the manuscript states 'All raw data underlying the findings reported within this manuscript are available at:
http://datadryad.org/stash/share/BD1LjvEVFKYEC_CiTiKaQ0uR8JoofTF_EFqSBVVUtyE.' Please provide the complete Data Availability statement in the submission form and ensure that it matches the one mentioned in the manuscript.

We have made all raw data available within the supplementary information. We have adjusted the manuscript to be consistent with this directive. See lines 560-562.
 
**Reviewer's Responses to Questions**

**Reviewer #1**: The manuscript by Skinner et. al. describes the effects of a sas4 conditional knockout on meiotic chromosome segregation during spermatogenesis and spermiogenesis. Sas4 conditional knockout male germ cells exhibit a failure to duplicate the centrioles in both meiosis I and meiosis II. Interestingly, despite a high percentage of meiosis II spermatocytes only having one centriole, these germ cells are still capable of forming a bipolar spindle and segregating chromosomes. The authors found that the acentriolar MTOCs contain MT-associated proteins. Even though chromosome segregation occurs in the absence of centriole duplication, the authors demonstrate that Sas4 and centriole duplication are required for accurate spermiogenesis.

Major comments:

1. Figure 3E. The authors show that centriole duplication fails in both meiosis I and meiosis II with ~60% of meiosis II spermatocytes with only 1 centriole per cell. The images show localization of both centrioles (CETN3) and spindle poles (alpha-tubulin). The representative image with only one centriole looks like it is still capable of forming a bipolar spindle. Is a bipolar spindle observed in all cells with only 1 centriole? Are monopolar spindles ever observed?

Yes, all *Sas4* cKO spermatocytes with a single centriole form bipolar spindle poles, we never see monopolar spindles. To emphasize this point, we have stated in the text that we only see bipolar spindles in *Sas4* cKO spermatocytes. Please see lines 209-212.

Related to these questions, it would be helpful to bring number of spindles and whether they are centriolar or acentriolar at the end of the paragraph on page 12 (~lines 205-213).

We further elaborated on this point as suggested. Please see lines 211-220.

2. On page 13 lines 244-245, the authors state that they wanted to examine whether oocyte acentriolar PCM proteins were present in the sas4 cKO acentriolar pole, yet both CEP192 and GCP2 are found in the centriolar based poles in control spermatogenesis. Either this sentence needs to be reworded (maybe to say lacking canonical PCM proteins) or oocyte specific PCM proteins needs to be used for the experiments.

Thank you for your suggestion, we have improved the clarity of this sentence to avoid confusion. Please see lines 245-259.

3. Figure 3D shows that a higher percentage of metaphase II cells have 1 centriole, which I presume would lead to one spermatid with 1 centriole and one with 0. But there are also ~20% with 2 centrioles, which should give rise to two spermatids each with 1 centriole. Can the authors speculate on why there is a higher percentage of round spermatids and elongating spermatids with 0 centrioles rather than 1 centriole (Figure 6B & 6C)?

Thank you for this question. The spermatids that inherit a single mature centriole are unable to successfully maintain attachment to the head of the spermatid because this connection is facilitated by the immature centriole. Attachment to the nucleus is initiated during the first four steps of spermiogenesis. This means that from step one onward during spermiogenesis, the association of the mature centriole with the nucleus could be lost in *Sas4* cKO spermatids. This dissociation results in an increase in round spermatids observed to harbor no centrioles. We elaborated on this point further in the manuscript. Please see lines 339-343.

4. In the discussion (page 18, lines 372-373), the authors state that a subset of MT-associated proteins are sufficient for chromosome segregation. This conclusion seems a little strong as sufficiency was not tested.

Thank you for your suggestion regarding providing additional evidence that chromosome segregation was successful. We have provided additional assessments of chromosome segregation during anaphase I and II and determined that there was no significant difference in observed chromosome lagging events between control and *Sas4* cKO spermatocytes. Please see lines 278-280 and Figure 6A and 6B.

Minor suggestions:

1. The yellow numbers indicating centriole numbers in figures 3 and 4 are hard to see. White numbers might provide more contrast with the black background.

Thank you for your suggestion. We have changed the color of the numbers on the figures to have higher contrast with the background. Please see Figures 3 and 4.

2. Figure 4E figure legend- need to define ncMTOC as this isn’t defined in the text until discussion of Figure 5.

We have added this information to our revised manuscript. Please see the Figure 4E legend (lines 948-949).

3. Define aMTOC on page 13 line 242.

aMTOC is defined earlier in the text. Please see line 150.

4. Very minor- Line 265 on page 14 change “an” to “a”. Should say which is a MT motor protein.

We have changed this in our revised manuscript. Please see line 271.

**Reviewer #2**: This is a well-written, clear and rigorous analysis of the defects observed when SAS4, a protein required for centriole biogenesis, is conditionally knocked out during spermatogenesis. In particular, the diagrams provided in the figures to orient the reader were very helpful. The authors report no defects in meiotic prophase but clear defects in centriole duplication. The defects in centriole duplication do not affect chromosome segregation in meiosis I or II because of the formation of noncentrosomal MTOCs but do affect later events in spermiogenesis, explaining the infertility defects they observe. I appreciated Figure 8, where the authors distinguished between the phenotypes observed in products of meiosis that did and did not receive a centriole. I have a few minor comments that might make this manuscript more widely accessible.

Line 155: “The large lumen diameter was due to the Sas4 cKO elongated spermatids not harboring flagella (Fig 1D).” Can the authors indicate in the control where the flagella are?

Thank you for your suggestion to indicate the flagella location with an arrow. Please see Figure 1D and the corresponding figure legend (lines 860-861).

Can the authors provide an image of 4 centrioles in SAS4 cKO? Are there any differences observed between this class in control and SAS4 cKO?

Thank you for the suggestion. We did not observe any differences between control and *Sas4* cKO metaphase spermatocytes when 4 centrioles are present. We have noted this in the text, please see lines 205-207. Because of this, we deemed it unnecessary to include another image panel.

I can’t see the maturation of the other parent centriole in the both control and cKO images in Figure 4C. I am much more convinced by the images in metaphase I. Would the pachynema images benefit from gray scale versions being available?

The images displayed in Figure 4C demonstrate pachynema cells that harbor only one mature centriole since that is what most of the population looks like at that time point. We have clarified this issue by also adding a CEP164 count on the respective images. Please see Figure 4B, C, D, and E and the corresponding figure legend (lines 934, 939, 944-945, and 950).

Can the authors provide more guidance in Figure 7 so that a reader unfamiliar with spermiogenesis can follow the phenotypes of the SAS4 cKO, especially in Figure 7E?

Thank you for the suggestion. We have provided more detail in the text of the figure legend. Please see Figure legend 7 (lines 1002-1005, 1016-1018, and 1031-1035).

**Reviewer #3**: This manuscript by Skinner et al. focuses on the role of SAS4, a centriolar protein, during spermiogenesis. They make a conditional knockout of Sas4 and study the consequences during meiosis and the formation of spermatids. They find that spermatozoa are not functional, causing infertility. Upon further analysis, meiotic prophase events seem normal, except that centrioles fail to duplicate during zygonema. Meiotic events still occur, but centrioles fail to duplicate again for meiosis II. They also lack centrosomal proteins, making them non-centrosomal MTOCs. They do have MT-associated proteins to coordinate spindle assembly. Despite the centriolar failure, the spermatids have chromatin (suggesting meiosis I and II occurred), but the major problems arise in formation of normal sperm. Of the steps of spermiogenesis, the defects start in step 9 with abnormal acrosome width, manchette length, perinuclear ring diameter, and the lack of a flagella.
This manuscript has interesting findings, that will contribute to the role of centrioles in spermiogenesis. While the results are primarily descriptive, they provide a foundation for future work. There are some issues that need to be considered:

1) The conditional knockout was made with the expression of CRE under the Spo11 promoter, which is turned on in preleptotene/ leptotene stage. There is no check that there is no protein production and that the protein made prior to Spo11 induction of CRE is turned over rapidly. The experiments in figure 2 are looking at events that occur early in prophase, leptotene and early zygotene. They do not observe defects with the cKO and interpret the results as Sas4 not having a role in those events, unlike Cep63. However, there are no experiments showing that the Sas4 protein is no longer present during those stages. The authors should determine if the protein is no longer present by performing immunostaining, or some other assay to look at levels of the Sas4 protein.

Thank you for your suggestion regarding the demonstration of SAS4 depletion in our *Sas4* cKO spermatocytes. Unfortunately, the antibodies against SAS4 that we tested were problematic with non-specific signals and could not be used to demonstrate protein depletion. Prior to submission, we had tested the Proteintech SAS4 antibody (11517-1-AP), but it exhibited broad cytoplasmic staining in both the control and *Sas4* cKO spermatocytes, indicating the antibody was not specific to the protein of interest. To attempt to address this comment, we ordered an additional antibody from Affinity (DF2312). Upon the recent arrival of this antibody (delayed due to the current, ever-changing US tariffs), we performed the same experiments, but we observed the same issue of broad cytoplasmic staining that was non-specific in both the control and *Sas4* cKO spermatocytes. Despite our efforts, we do not believe the demonstration of protein depletion should be a significant concern, however, because of the specific phenotypes we observe and report relating to centriole duplication failure. Furthermore, this floxed mouse model has already been well-established as a robust model. We and others have previously characterized the Sas4 cKO in other studies, and is a commercially available line from the International Mouse Phenotyping Consortium (<https://www.mousephenotype.org/data/genes/MGI:2684927>). Please see the following references as examples.

- <https://pubmed.ncbi.nlm.nih.gov/24706806/>
- <https://pubmed.ncbi.nlm.nih.gov/30626697/>
- <https://www.embopress.org/doi/full/10.15252/embj.2020106118>

Additionally, the *Spo11*-Cre mouse line that we utilized to generate the *Sas4* cKO during early meiosis in spermatocytes is also a well-established mouse line that we have successfully used in-house with *Sas4* and other cKO alleles with efficient Cre excision. Please see the following references for examples.

- <https://pubmed.ncbi.nlm.nih.gov/23468651/>
- <https://www.embopress.org/doi/full/10.1038/s44319-024-00187-6>
- <https://www.embopress.org/doi/full/10.15252/embr.202051023>
- <https://pubmed.ncbi.nlm.nih.gov/30281394/>

We’d also like to point out that studies have been published that assess the *Sas4* cKO mouse model but do not assess SAS4 protein depletion. Please see the following reference as an example from EMBO J.

- <https://www.embopress.org/doi/full/10.15252/embj.2020106118>

**In summary**, we regret that our efforts to show SAS4 protein depletion were not fruitful, but we believe given that: (1) SAS4 is a centriole component, (2) we see inability for the spermatocytes to build new centrioles in the *Sas4* cKO condition, (3) because of the centriole duplication deficiency, the *Sas4* cKO male animals are infertile due to aberrancies during spermiogenesis, and (4) the *Sas4* cKO allele and Cre driver have both been previously well characterized for their KO efficiency – We strongly believe this provides sufficient support for publication.

2) The authors mention that meiosis II was successfully completed, but did they track whether chromosome segregation occured normally?

Thank you for this comment about chromosome segregation assessment. We have provided additional assessments of chromosome segregation during anaphase I and II and determined that there was no significant difference in observed chromosome lagging events between control and *Sas4* cKO spermatocytes. Please see lines 278-280 and Figure 6A and 6B.

3) In Fig 7, multiple steps are represented in one image (eg. steps 1-7). Is this because you cannot tell the difference between steps 1-7? If you can tell the difference, it may be better to state the actual step imaged instead of lumping them all together.

We chose to include steps 1-7 together because it is difficult to accurately distinguish between them with our methods of assessment. Furthermore, we do not observe any differences between control and *Sas4* cKO spermatids during steps 1 to 8 that would require us to distinguish between these steps (Figure 7). It is common practice to group steps of spermiogenesis together for reporting purposes, depending on the assessment needed. Please see the following references as examples of this point:

- <https://www.pnas.org/doi/10.1073/pnas.2313787120>
- <https://www.nature.com/articles/s41598-024-84006-x>
- <https://pubmed.ncbi.nlm.nih.gov/39732242/>

4) In Fig 7H-I, what is the inset box in the left corner showing? The figure legends says DAPI, but is it the entire structure? The other inset boxes zoom into smaller structures.

Yes, the inset images do show the entire DAPI signal for each respective spermatid. The purpose of the grey inset in the lower left corner is to highlight DNA morphology. We explain this in the Figure legend, lines 1056 - 1059.
